# Disruptive Innovation Can Prevent the Next Pandemic

**DOI:** 10.3389/fpubh.2015.00215

**Published:** 2015-09-23

**Authors:** Affan T. Shaikh, Lisa Ferland, Robert Hood-Cree, Loren Shaffer, Scott J. N. McNabb

**Affiliations:** ^1^Public Health Practice, LLC, Atlanta, GA, USA; ^2^Northrop Grumman Corporation, Falls Church, VA, USA; ^3^Emory University, Atlanta, GA, USA

**Keywords:** disruptive innovation, public health surveillance, one health, public health surveillance strengthening, e-Surveillance, public health informatics

## Abstract

Public health surveillance (PHS) is at a tipping point, where the application of novel processes, technologies, and tools promise to vastly improve efficiency and effectiveness. Yet twentieth century, entrenched ideology and lack of training results in slow uptake and resistance to change. The term *disruptive innovation* – used to describe advances in technology and processes that change existing markets – is useful to describe the transformation of PHS. Past disruptive innovations used in PHS, such as distance learning, the smart phone, and field-based laboratory testing have outpaced older services, practices, and technologies used in the traditional classroom, governmental offices, and personal communication, respectively. Arguably, the greatest of these is the Internet – an infrastructural innovation that continues to enable exponential benefits in seemingly limitless ways. Considering the Global Health Security Agenda and facing emerging and reemerging infectious disease threats, evolving environmental and behavioral risks, and ever changing epidemiologic trends, PHS must transform. Embracing disruptive innovation in the structures and processes of PHS can be unpredictable. However, it is necessary to strengthen and unlock the potential to prevent, detect, and respond.

## Introduction

Fifty-two years ago, Alexander Langmuir articulated our modern understanding of public health surveillance (PHS) – the systematic collection, consolidation and evaluation, and dissemination of data (1). In this workflow process, public health provides epidemiologic intelligence to assess and track conditions of public health importance, define public health priorities, evaluate programs, and conduct public health research (2). However, amid this rapidly changing world, PHS has remained sluggish and hindered by the impediments of siloed, vertical (outcome-specific) systems, inadequate training and technical expertise, different information and communication technology (ICT) standards, concerns over data sharing and confidentiality, poor interoperability, and inadequate analytical approaches and tools (3–7).

Gaps and impediments in PHS have become increasingly evident to the world in the wake of the largest Ebola epidemic ever – in which these challenges impacted our ability to prevent, detect, and respond. Under the looming threat of MERS-CoV, leishmaniasis, influenza, multidrug-resistant tuberculosis, and plague, the global public health community now realizes the urgent need to address shortcomings in PHS. Properly preparing for the next major outbreak hinges on our willingness to transform; the consequences of not doing so are dire.

Transforming PHS to meet the needs of the twenty-first century requires novel approaches. A helpful concept to understand and chart this future is *disruptive innovation* – a term first introduced by Clayton Christensen to describe innovations in technology and processes that disrupt existing markets (8). Disruptive innovations occur when advances in technologies or processes create markets in existing industries. This differs from sustaining innovations, where existing practices are incrementally improved to meet the demands of existing customers; in contrast, newly introduced innovations with disruptive potential (typically unrefined, simple, and affordable in character) target lower-end market needs or create entirely new market segments. As sustaining innovations improve disrupting technologies or processes, these new innovations will meet increasingly greater needs, capture greater market share, and eventually reshape the industry. Christensen uses the example of increasingly smaller disk sizes in the hard disk drive industry, the introduction of hydraulic technology in the mechanical excavator industry, and the rise of minimills in the steel industry to demonstrate the impact of disruptive innovations (8). Here, we describe the need for disruptive innovation in PHS and identify opportunities for disruption in PHS structures and processes.

## Disruptive Innovation in Public Health Surveillance

To fulfill the Global Health Security Agenda and improve population health, PHS requires systematic improvements in planning and system design, data collection, data management, analysis, interpretation, dissemination, and program application. Numerous opportunities for disruption may affect any one of these activities. Taking stock of the challenges facing PHS in 2012, Thacker et al. described six concerns (9):

- complicated and heterogeneous lexicon
- expanding global surveillance networks to address evolving needs
- inadequate use of ICT tools
- lack of proper and comprehensive workforce development
- inconsistent data access and use
- poor data management, storage, and analysis practices

Disruptive technology can overcome these challenges. One example is the incorporation of digital tools to create electronic-based surveillance (e-Surveillance) – an ongoing disruption of paper-based methods. Adoption of the Internet enforced the need for standardization of vocabularies and opened doors for greater connectivity of local and global networks. Additionally, online training programs, e-Universities, and distance learning methods such as massive open online courses have opened unprecedented educational opportunities. Applications of ICT tools, such as Epi Info™ at the U.S. Centers for Disease Control and Prevention (CDC), have greatly improved data access, management, storage, and analysis practices. According to a 2012 Council of State and Territorial Epidemiologists assessment survey, health departments across the United States currently use a variety of notifiable diseases surveillance systems including custom-built systems, commercially available systems (e.g., Massachusetts Virtual Epidemiological Network, Scientific Technologies Corporation, Atlas, and Trisano), and the CDC National Electronic Telecommunications System for Surveillance (NETSS). As one of the first electronic systems to be developed in the early 1990s, NETSS uses a case-based structure (10, 11). However, as NETSS is restricted in functionality, a number of states continue to transition to the person- and standards-based National Diseases Surveillance System (NEDSS), a process that has been ongoing since 1998. NEDSS aims to integrate HIV/AIDS reporting systems, vaccination programs, and tuberculosis and other infectious disease tracking programs (10). Prior to NEDSS, these compartmentalized systems were isolated from one another due to differing data standards, legacy systems, and lack of tools for information exchange (10).

## Disrupting Public Health Surveillance Structures

Demonstrated by the recent Ebola outbreak, the structure of PHS, including the various governance and collaborative frameworks guiding prevention, detection, and response, needs modernization. Foremost are the World Health Organization’s (WHO) International Health Regulations (12), last revised in 2005. Meant to act as a safeguard against health threats by strengthening a country’s capacity to detect, assess, respond, and report public health emergencies, its implementation where it is most needed falls short of achieving its purpose. One WHO report assessing implementation of the IHR (2005) in 2013 showed the African region to be well below global averages across all attributes measured, with no African state reporting full implementation (13, 14).

In the wake of the 2014 Ebola, successful compliance with the IHR (2005) requires much greater sustained investment in implementation (15). However, to meet evolving needs, this investment must be coupled with additional public health surveillance strengthening (PHSS). In early 2014, the United States of America in collaboration with 28 countries, the WHO, the Food and Agricultural Organization of the UN (FAO), and the World Organization for Animal Health (OIE) set forth to advance the IHR (2005) with the launch of the Global Health Security Agenda (16). The Agenda provides a renewed attempt to provide a framework and path with targets and milestones to accelerate progress in strengthening PHS.

### New frontiers

Public health surveillance strengthening requires disruption of governance and collaboration. While historically infectious disease centric, the scope of PHS has vastly expanded over recent years to include surveillance of chronic conditions and occupational hazards among many other public health issues. Furthermore, prevention, detection, and response are not restricted to national or regional health departments, as seen with the emergence of participatory PHS. Rather PHS is multisectoral, multilateral, and bidirectional. Recent years have given rise to new governmental, non-governmental, for-profit, and academic actors working at various levels (e.g., international, national, regional, and local) to fill gaps and meet needs while increasingly engaging the public.

With growing immediacy of the interaction between humans and animals, *One Health* has also emerged as a prerequisite for PHSS. With at least 60% of emerging and reemerging human infectious diseases being zoonotic, *One Health* unites human, veterinary, and environmental health disciplines for a more holistic approach to address the challenges we face (17). Leveraged for PHSS, *One Health* is a disruptive force in how we collaborate to prevent, detect, and respond to public health emergencies. A fully realized and integrated model of *One Health* PHS creates a proactive shift in prevention and response to the source – a disruption of existing, reactive PHS moving from outbreak to outbreak. However, achieving *One Health* PHS requires overcoming barriers. A review of *One Health* adoption by Uchtmann et al. highlighted underserved populations, professional barriers, incompatible vocabularies, sequestration of data, and territorial borders as impediments (18).

As *One Health* gains acceptance, the public health workforce will require interdisciplinary approaches to training. The CDC’s Epidemic Intelligence Service (EIS) pioneered field epidemiology training. Accepting physicians, nurses, veterinarians, and persons with health science doctorates into the program indicates the growing acceptance of multidisciplinary PHS. Internationally, field epidemiology training programs (FETPs) offer robust solutions to the training needs of the public health workforce. The 55 accredited FETPs across the globe link to regional networks and the umbrella network known as the Training Programs in Epidemiology and Public Health Interventions Network (TEPHINET). They provide competency-based apprenticeships in applied epidemiology. FETP trainees have first-hand experiences responding to numerous cross-border and global public health investigations including disaster responses, non-communicable diseases, and emerging or reemerging infectious disease threats. Together the EIS and FETP programs have trained over 6,980 public health professionals (19). This represents a critical resource for a poorly staffed workforce. With a trained workforce, innovative programs such as the U.S. Agency for International Development’s Emerging Pandemic Threats program will have expertise to draw from across the animal and human health sectors to inform their PREDICT, PREVENT, IDENTIFY, and RESPOND projects and help build regional, national, and local *One Health* capacities for early disease detection, laboratory-based disease diagnosis, rapid response and containment, and risk reduction (20).

To accomplish robust IHR (2005) implementation and enhanced global health security, PHSS requires a well-trained public health workforce focused on *One Health* prevention in surveillance, epidemiology, laboratory, communications, and outbreak investigation. Developed countries must think globally and invest in developing countries’ infrastructures and establish integrated PHS with proactive collaborative agreements to respond to public health emergencies. Enhanced governance frameworks, such as the Global Health Security Agenda, are critically important. Nigeria’s prompt response and containment of the 2014 Ebola epidemic has been attributed to preexisting structures like a public health emergency operations center and available FETP trained epidemiologists, both targets of the Global Health Security Agenda (21).

## Disrupting PHS Processes

Globalization has drastically changed our interactions with the biological world. A novel pathogenic infection discovered in one part of the globe can be easily carried thousands of miles away in a single day. Yet our implementation of the processes of PHS, including various advances in informatics and analytical tools, remains underutilized. As an example, the Integrated Disease Surveillance and Response (IDSR) regional framework, adopted in 1998 by the African regional office for the WHO, is a novel attempt at strengthening PHS capabilities at all levels in Africa (22). However, as recent studies reveal, this paper-based framework lacks timely reporting of PHS data and has been reported to be generally inefficient, error-prone, incomplete, and untimely (23). Disruptive innovation of the informatics and analytics used by PHS can address these gaps and impediments and improve IDSR. Developments in electronic health records, interoperability, information exchange, public information sharing, decision support, and cloud technologies are pushing ICT capabilities faster than PHS can evolve for the prevention, detection, and response. Once these advances are implemented, e-Surveillance can be fully realized and leveraged.

### New opportunities

Following sustained efforts toward PHSS, PHS can leverage novel, disruptive e-Surveillance approaches using informatics and analytics. Use of improved informatics techniques have been shown to improve completeness and timeliness of PHS data, but this depends critically on uniform standards of reporting, efficient workflow processes, and the willingness of practitioners to adopt disruptive technologies and processes (24, 25).

Disruptive innovations are not necessarily the most advanced technologies, but more often novel combinations of existing technologies or processes, offering simple and affordable alternatives. An emerging example of this sort of disruptive innovation in PHS is the emergence of participatory PHS with geographical information systems (GIS). Coupled with increasing availability and access to the Internet and mobile-based technologies, participatory PHS has also emerged in recent years as an innovative method of engaging the public and collecting regular, voluntary syndromic data (26). Examples of these PHS systems include ProMED-mail, Influenzanet, FluTracking, Reporta, Flu Near You, Dengue na Web, SaludBoricua, TuAnalyze, and Ushahidi. Common among these new PHS tools is an ability to aggregate, analyze, and visualize data in charts and maps in near real time while being freely accessible and easy to use.

Innovations in information aggregators for PHS leverage advances in the Internet and GIS. HealthMap, founded in 2006 by researchers at Boston Children’s Hospital, combines various data from online news aggregators, eyewitness reports, expert-curated discussions, and validated official reports to map a unified and comprehensive view of the current global state of infectious diseases (26). While the free or low-cost nature is attractive and beneficial, the acceptability of these programs into mainstream systems depends greatly on their ability to enhance already existing data streams, something yet to be realized (27). As reported by Velasco et al., a number of vital issues must be addressed prior to full integration including time-consuming and costly collaboration with statisticians, Internet and media experts, and computer scientists to work on components of data acquisition, data processing and filtering, personalization of results, and automation and verification of data (28). However, even with full integration, PHS must find a balance in supplementing these new technologies with existing PHS systems with official detection, verification, and validation responsibilities, using confidential sources. This underscores the need for PHSS before implementation of e-Surveillance.

The increasing prevalence of mobile and wireless technologies, recognized for their potential impact on health by the WHO in 2011, also offers a unique opportunity for disruption of PHS processes (29). With rapid technological development, falling market prices, increasing network coverage, and explosive user growth, the developing world has the greatest to gain from the implementation of mobile and wireless health technologies (30). Mobile networks are particularly valuable when, considering in some parts of the world, mobile penetration has outpaced other advanced communication technologies, extending far beyond the electrical grid and health infrastructure in some instances (31).

However, despite pervasive attributes, technological inequalities remain an important consideration. Among cell phone users today in the United States, African Americans and Hispanics are more likely to look up health information using a mobile device than are White non-Hispanics (32). In Brinkel et al.’s review of mobile health practices for PHS in Sub-Saharan Africa, PHS with real-time and validated data was strongly needed to strengthen disease monitoring capacity. However, mobile phone-based projects in PHS continue to be small-scale and fragmented (31). The success of mobile health projects generally correlates with their accessibility, acceptance, adaptation to local contexts, cost of the technology, stakeholder collaboration, and government involvement (33).

## Discussion

The desire for comprehensive PHS with interoperable electronic systems and data captured from many sources and across many diseases is not new but is still far from being realized (34). Disrupting the structures and processes of PHS with novel technologies and processes will help achieve this vision. Strong and sustained investments in PHSS leveraging opportunities in One Health and applied epidemiology training programs will bolster the structures for prevention and response to public health emergencies. As PHSS takes hold, innovative approaches toward e-Surveillance, like participatory systems and mobile health, can be leveraged to drastically improve detection of public health emergencies. The developing world, with limited resources and infrastructure capabilities, and less access to higher market end traditional PHS systems, stand to benefit the greatest from these disruptions. Together, disruptions in structures and processes leading to PHSS and e-Surveillance promise to transform current practice; a new vision emerges where the PHS workforce implements the latest technologies and processes, and the information required to make informed decisions is available when it is needed, where it is needed.

## Conflict of Interest Statement

The authors declare that the research was conducted in the absence of any commercial or financial relationships that could be construed as a potential conflict of interest.
